# Melatonin Treatment in Rams and Their Replacement with Novel Treated Rams Advance First Lambing and Increase Fertility in Sarda Ewe Lambs

**DOI:** 10.3390/ani11051227

**Published:** 2021-04-23

**Authors:** Giovanni Cosso, Sebastiano Luridiana, Luisa Pulinas, Giulio Curone, Giulia Pich, Vincenzo Carcangiu, Maria Consuelo Mura

**Affiliations:** 1 Department of Veterinary Medicine, University of Sassari, 07100 Sassari, Italy; gicosso@uniss.it (G.C.); sluridiana@uniss.it (S.L.); luisapulinas@hotmail.it (L.P.); g.pich93@gmail.com (G.P.); endvet@uniss.it (V.C.); 2 Department of Veterinary Medicine, University of Milan, 26900 Lodi, Italy; giulio.curone@unimi.it

**Keywords:** melatonin implants, ram replacement, puberty, ewe lambs, sarda breed

## Abstract

**Simple Summary:**

The goals of this study were to advance first mating in ewe lambs and to shorten the period ranging from weaning to first lambing. Sarda ewe lambs (n = 400) were separated into four groups of 100 and exposed for a 50-day breeding period to fertile, adult rams as follows: (1) RMR (Rams–Melatonin–Replacement) group: exposed to melatonin-treated rams which were replaced every 10 days; (2) RM (Rams–Melatonin) group: exposed to melatonin-treated rams which were not replaced; (3) RCR (Rams–Controls–Replacement) group: exposed to untreated rams which were replaced every 10 days; and (4) RC (Rams–Controls) group: exposed to untreated rams which were not replaced. In each group, lambing dates, fertility rate, litter size, and distance in days from ram introduction to lambing (DRIL) were recorded. The RMR group showed the highest fertility rate, whilst shorter DRIL and higher number of ewes that lambed in a shorter time frame were recorded both in RM and RMR groups, compared to controls. The findings highlighted that melatonin treatment in rams and their replacement allowed advancing first mating, increasing fertility rate, and improving lambing concentration.

**Abstract:**

This study aims to find reliable strategies for advancing first mating and shortening the period from weaning to first lambing in ewe lambs. Sarda ewe lambs (n = 400) were selected from two farms and allocated into four separated groups of 100, all of which were exposed to fertile, adult rams over the course of a 50-day breeding period. The first treatment group (RMR) was exposed to four melatonin-treated rams which were replaced every ten days, whilst the second treatment group (RM) was exposed to four melatonin-treated rams which were not replaced. Alternatively, the first control group (RCR) was exposed to four untreated rams which were replaced every ten days, whilst the second control group (RC) was exposed to four untreated rams which were not replaced. In each group, lambing dates, fertility rate, litter size, and distance in days from ram introduction to lambing (DRIL) were recorded. The highest fertility rate was recorded in the RMR group (*p* ≤ 0.05). Shorter DRIL (*p* ≤ 0.01) and higher lambing concentrations were recorded in the RM and RMR groups as compared to the controls. The findings indicate that melatonin treatment of rams and their replacement at 10-day intervals results in earlier onset of first mating, increased fertility rate in ewe lambs, and a higher number of ewes that lambs in a shorter time frame.

## 1. Introduction

The shortening of the time period from weaning to first lambing has a key role in sheep farming management improvement. Advancing the onset of first mating, therefore decreasing the age at which it first occurs in young females, which lamb at 8–9 months of age, offers an array of economic benefits, including the reduction of maintenance costs for replacements and the increase of lamb crop per female during her reproductive life [1]. The duration of the reproductive life of ewe lambs is influenced by several environmental and genetic factors [2]. Among environmental cues, the season greatly influences reproduction and puberty onset in sheep owing to photoperiodic change, that is, a change in the duration of sunlight hours within a day [3]. At temperate latitudes, short photoperiods stimulate the hypothalamus–pituitary axis through the circadian secretion pattern of melatonin by the pineal gland resulting in the stimulation of reproductive activity [4]. Due to this photoperiodic effect, winter- and spring-born ewe lambs tend to reach puberty during the following autumn (aged 7–9 months), whilst those born in autumn experience a delay in the onset of puberty until the next autumn (aged approximately 1 year) [5,6]. As Sarda sheep produce large milk yields, their reproductive functions remain poor for at least 2 months after lambing, leading to a delay in the second lambing period [7,8,9]. Several studies have focused on the beneficial effect of male presence on both reproductive recovery in adult ewes and advancing puberty in ewe lambs [10,11,12]. This effect is more proficient during rams’ full reproductive activity periods, which are modulated by the photoperiodic trend.

Shorter photoperiods result in higher semen quality and testosterone levels, whereas longer photoperiods between late winter to mid-summer result in lower semen quality and declines in sexual behavior [13]. Therefore, past studies have documented that melatonin treatment, which mimics short photoperiods, results in improved semen quality and sexual behavior (flehmen, ano-genital sniffing, attempted mounting) [14]. After subcutaneous implants, the melatonin concentration in blood is similar to the normal night-time levels for more than 70 days [15].

Consequently, the consistent presence of sexually active rams, alongside a set of socio-sexual signals, is a compelling stimulus that is able to partially counteract the photoperiodic control of seasonal reproduction in ewes [16]. The ovulatory response to ram effect is known to be positively correlated with the duration of ram presence [17]. As a result, this increases the number of matings, and consequently of lambings, in a shorter period than among the ewes isolated from rams prior to mating [18]. In order to avoid the habituation and, therefore, refractoriness to the ram stimulus in the ewes, the replacement of familiar rams with novel rams is found to be a useful strategy to improve reproductive response [19,20].

The purpose of this study was to evaluate whether melatonin treatment of rams alone, or coupled with their replacement every 10 days with novel rams could be reliable strategies for: (1) advancing first mating and (2) shortening the period from weaning to first lambing in sheep.

## 2. Materials and Methods

### 2.1. Animals

The animals in this study were managed and treated by the farm’s veterinarian following the guidelines of the Animal Welfare Act, and were under the control of the National Health Veterinary Service of Italy. The treatments made in this study were commonly used techniques performed on sheep farms to improve the flock’s reproductive performance. The reproductive activity in Sarda ewes is influenced by the Mediterranean climate, which determines herbage availability usually in autumn and mostly in the spring season. In the typical Sardinian farming system, adult ewes are mated from May to July so that lambing can occur in autumn, in order to exploit the availability of the natural green grass and to ensure a longer lactation period [21]. Instead, young ewes usually lamb between January and March, depending on the period of their birth and of their body development [7]. This study was conducted during the breeding season in two farms, situated approximately 8 km apart in North Sardinia, Italy (40° 48’ N, 8° 16’ E); each raising approximately 1000 Sarda breed sheep. The farms were under the same pedoclimatic condition and had the same feeding and sanitary management, as they were served by the same veterinarian and nutritionist. In each farm, the ewe lambs were weaned between 30 and 40 days of age. In the first month after weaning they were fed with a commercial weaning food and had water *ad libitum* and free access to alfalfa hay (*Medicago sativa*). After this period, the ewe lambs received approximately 400 g per day of concentrated feed for growth, they grazed on legumes and gramineous grass during the day, and had free access to hay and water in the fold throughout the night.

### 2.2. Groups Formation and Experimental Treatments

Upon weaning, the ewe lambs from each farm were housed together in one of the farms used for the study to receive the same managerial treatment. On 10 July 2017, 400 ewe lambs born from a single lambing between 1 November 2016 and 30 November 2016 were chosen. All ewe lambs weighed a minimum of 28 kg, which is higher than 60% of mean adult ewes liveweight (42–45 kg). The animals were identified with an electronic rumen bolus, which was read through the appropriate transponder (Allflex RS420 Allfelx Livestock Intelligence, portable stick reader, Palmerston North, NZ) and registered in order to avoid recognition error.

On 15 June 2017, 24 fertile, adult rams (aged 2.5–6.5 years) from both farms were treated with three melatonin implants—Melovine^®^, Ceva Salute Animale, Agrate Brianza, MB, Italy—each containing 18 mg melatonin, in the left retro-auricular region. A second group of 24 fertile adult rams (aged 2.5–6.5 years) from both farms remained untreated. The health of the rams was confirmed by the veterinarian and they were assumed to be fertile as they had produced offspring in previous breeding seasons.

The ewe lambs were distributed into four groups, named RMR(Rams–Melatonin–Replacement), RM (Rams–Melatonin), RCR (Rams–Controls–Replacement), and RC (Rams–Controls), each comprising 100 subjects, based on their exact date of birth and liveweight (Table 1). Following group formation, the four groups remained separated for the entirety of the study but were kept under the same feeding and managerial conditions. On 20 July 2017, the ewe lambs in all the experimental groups were exposed to rams. The first treatment group was exposed to four melatonin-treated rams, which were replaced by four novel melatonin-treated rams every ten days (RMR). Therefore, the ewes in this RMR condition were exposed to a total of twenty melatonin-treated rams over the course of the 50-day breeding period. The second treatment group of ewe lambs was exposed to four melatonin-treated rams (RM) and exposure to the same rams was continued over the course of the 50-day breeding period. On the other hand, the first control group of ewe lambs was exposed to four untreated rams, which were replaced by four novel untreated rams every ten days (RCR). Therefore, the ewes in this RCR condition were ewes that lambed exposed to a total of twenty untreated rams over the course of the 50-day breeding period. The second control group of ewe lambs was exposed to four untreated rams (RC) and exposure to the same rams was continued over the course of the 50-day breeding period. Rams in all conditions were provided with marker harnesses, which were changed every ten days, to allow the daily registration of mating.

Trans-vaginal echographic pregnancy diagnoses were carried out from 45 days after ram introduction until 45 days after ram removal. From 150 to 200 days after ram introduction, lambing dates and the number of newborn lambs were recorded. From the recorded data, fertility rate, the number of ewes that lambed per ewe exposed to the ram, mean litter size, the number of newborn lambs per ewe lambing, and the distance in days from ram introduction to lambing (DRIL) were calculated. The onset of reproductive activity in the different groups was calculated through DRIL. Once lambed, the females were considered adult ewes and no longer ewe lambs.

### 2.3. Statistical Analysis

R statistical software (Version 4.0.4 R Core Team 2021 R: A language and environment for statistical computing. R Foundation for Statistical Computing, Vienna, Austria.; https://www.R-project.org/, accessed on 15 January 2021) was used to analyze the associations between the two variables—melatonin treatment and male replacement—and the reproductive activity, measured as fertility rate, litter size, and DRIL. To compare the fertility rate and the number of ewes that lambed in 10-day periods (from 150 to 200 days after ram introduction) among the different groups, a Chi-square test was used. To analyze the DRIL and litter size, the following linear model was performed:

*y_ijkmn_* = *µ* + *T_i_* + *R_j_* + (*T_i_R_j_*)+*F_k_* + *S_m_* + *e_ijkmn_* (1)

where *y_ijkmn_* is the trait measured for each animal, *µ* is the overall mean of the studied ewes, *T_i_* is the fixed effect of the melatonin treatment (2 levels, treated and untreated), *R_j_* is the effect of the ram replacement (2 levels, replaced and not replaced), *T_i_R_j_* is the interaction between melatonin treatment and ram replacement, *F_k_* is the fixed effect of the farm (2 levels, 1 and 2 farm), *S_m_* is the random effect of the ram (48 rams), and *e_ijkmn_* is the random residual effect of each observation. Multiple comparisons of the least square means were then performed using Tukey’s method (library Agricolae, R package version 1.3-1. Statistical Procedures for Agricultural Research. https://CRAN.R-project.org/package=agricolae, accessed on 7 January 2021). Data on DRIL and litter size are expressed as least square means ± SEM. An ANOVA was performed to distribute weight and age of the chosen ewe lambs in the groups. *p* ≤ 0.05 was considered statistically significant.

## 3. Results

In the present study, the ewe lambs’ body weight at the moment of ram introduction was more than 60% of the adult weight. Age and weight did not differ significantly (*p* > 0.05) across the groups of ewes (Table 1).

**Figure 1:**
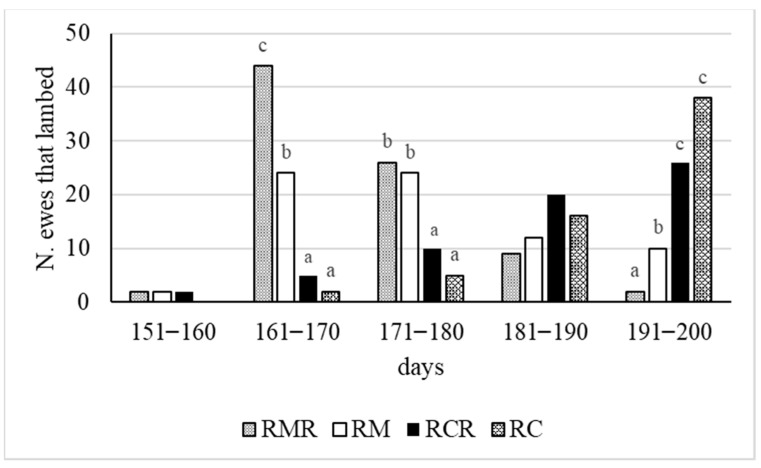
Number of ewes that lambed from 150 to 200 days (in 10-day periods) after ram introduction in the observed groups. The number of ewes that lambed in each 10-day period was compared by a Chi-square test. RC = ewes mated with untreated control rams; RM = ewes mated with melatonin-treated rams; RMR = ewes mated with rams treated with melatonin and replaced every 10 days; RCR = ewes mated with untreated control rams replaced every 10 days. Different letters (a, b, c) indicate a difference for *p* ≤ 0.01.

The lambing trend shown in Figure 1 was similar to mating and pregnancy trends (results not shown in Figure 1).

A difference of approximately 3% was reported between the number of ewes mated and pregnant, whereas a difference of approximately 2% was reported between pregnant and lambed (Table 2).

The interaction between treatment and male replacement for DRIL was statistically significant (*p* ≤ 0.01). Furthermore, DRIL was statistically different between ewes exposed to melatonin-treated rams and those exposed to untreated rams (*p* ≤ 0.01). Likewise, the effect of male replacement was significant for DRIL (*p* ≤ 0.05). Indeed, ewes exposed to rams treated with melatonin and subjected to male replacement (RMR) showed a lower DRIL compared to those exposed to untreated rams replaced every 10 days (RCR) (*p* ≤ 0.01) (Table 3). Furthermore, animals treated with melatonin (RM) showed a lower least square mean for DRIL compared to untreated animals (RC) (Table 3).

The RMR group displayed the most mating at approximately 19 days following ram introduction. The RM group did not display a significant mating peak, however, a higher occurrence of mating was recorded between 18 and 30 days following ram introduction. In both the RC and RCR groups, the number of matings peaks around 44 days after ram introduction. The RMR group showed a higher number of ewes that lambed than the other groups (*p* ≤ 0.05) and the RM group showed a higher number of ewes that lambed than RC and RCR groups (*p* ≤ 0.05) (Table 2). Litter size was not significant for any factor of the model (*p* > 0.05) (Table 3).

Lambing registered from 150 to 200 days after ram introduction exhibited a different number of ewes that lambed in the four groups (Figure 1). The RMR group exhibited an increase in the number of ewes that lambed in the first 20 days of lambing (*p* ≤ 0.01) (Figure 1). At 180 days after ram introduction, the RM and RMR groups showed a greater occurrence of lambing compared to the RC and RCR groups (*p* ≤ 0.01) (Figure 1).

## 4. Discussion

This study found that melatonin treatment in rams and their replacement with novel melatonin-treated rams at 10-day intervals influenced first mating and lambing in Sarda ewe lambs. The use of melatonin-treated rams alone, or accompanying replacement with novel rams, was associated with the highest fertility rate and advanced onset of first mating within the subject pool. These findings support past research as melatonin treatment and photoperiodic effects were also associated with improvements in ram reproductive performance in Rasa Aragonesa rams, and earlier onset of puberty in Rasa Aragonesa ewe lambs [12,21]. The reduction in the age at which first lambing occurred, as observed in this study, is also comparable to that found in previous experiments with melatonin-treated Sarda ewe lambs [22,23].

There is a consensus in the literature that increases in photoperiods from late winter to mid-summer at temperate latitudes result in lower semen quality and a decreased libido in rams [14]. Melatonin treatment in rams allows the imitation of short photoperiods, thus improving semen quality, and increasing testosterone and LH secretion and testicular weight [24]. Moreover, melatonin receptors have also been found in different parts of the ram reproductive system [25], thus confirming the importance of this indolamine in the testis functionality [26]. These considerations can explain how melatonin affects ram sperm functionality directly, controlling different phases such as capacitation and reducing apoptosis and oxidative stress [27]. Additionally, melatonin concentration, testosterone levels, and antioxidant enzyme activity in the seminal plasma have been known to vary seasonally [28]. The antioxidant capacity of melatonin in ram spermatozoa could explain its anti-apoptotic action, since high levels of reactive oxygen species (ROS) lead to cell death [29,30,31]. Melatonin treatment in Raza Aragonesa rams has been seen to cause an immediate increase of melatonin in the seminal plasma leading to differences in ram sperm quality and fertility [32]. Therefore, in the present study, the melatonin treatment may have led to an increase in testosterone and a decrease in ROS in the rams’ reproductive tract, thus leading to improved reproductive performance. Improved ram reproductive efficiency enhanced fertility in ewes exposed to melatonin-treated rams and advanced their first mating.

The present study, for the first time, analyzed the effect of melatonin treatment in rams combined with ram replacement on the time of first mating of ewe lambs. Previous research found that in adult ewes, weekly ram replacement alone could increase fertility rate and advanced reproductive resumption in spring [9]. The results of this study also agree partially with what has been reported in English crossbred sheep (Scottish Blackface × Bluefaced Leicester) wherein the replacement of rams every 15 days improved reproductive activity [20]. In fact, in the present study, ram replacement alone did not increase the fertility rate. This difference with mature ewes could be attributed to the fact that reproductive activity in adult ewes is more easily stimulated compared to ewe lambs [33]. Thus, it can be hypothesized that exposure to melatonin-treated rams, coupled or not, with their replacement every 10 days resulted in a stronger stimulus for reproduction for the ewe lambs than the ram novelty alone. Furthermore, in addition to the highest fertility rate, more ewes exposed to RMR rams mated in the first 20 days than those exposed to the other rams. This trend within the RMR group was further confirmed by the shortest DRIL and by the number of the total lambing from 161 to 180 days after ram introduction. Hence, the “novelty effect”, as also hypothesized by Hawken and Beard [20], could be the reason for a stronger stimulus at the hypothalamus-pituitary-gonadal axis level, thus promoting a reduction of the age at which the onset of reproductive activity occurred in ewe lambs. Furthermore, the differences found in the fertility rate and in the age at which onset of reproductive activity occurred between the RMR and RCR groups could be ascribed to melatonin treatment in the RMR rams, as they suggest that the treatment could have promoted RMR rams to be more sexually active. Therefore, the novelty effect in the ewes exposed to the RMR rams was stronger than in the RCR group. The introduction of rams in the flock results in an increase of LH basal secretion in ewes and a subsequent LH peak within 20 min, thus triggering reproductive activity [34]. These factors suggest that the melatonin-treated rams may have provided ewes with a stronger stimulus, therefore leading to the following events: silent estrous with the development of a normal corpus luteum with a normal length of the luteal phase; followed by another ovulation, this time with estrus approximately 20 days following ram introduction, as also reported by Martin et al. [35]. Untreated rams may have provided low levels of stimulation for ewes, thus leading to a silent estrous that developed a subnormal corpus luteum, had a shorter life span, and regressed at approximately a week. In fact, the normally high luteal-phase progesterone concentrations that suppress GnRH secretion to low levels may not appear during the first reproductive cycle [36]. This subnormal corpus luteum presumably caused a short cycle which was unable to stimulate the hypothalamus-pituitary axis, as also documented by Rosa and Bryant [24], producing another silent estrous and finally a fertile estrous at approximately 30–40 days following ram introduction. Therefore, the greatest reproductive efficiency in the RMR and RM groups may have been enabled via the higher sexual activity of rams. Melatonin treatment in rams stimulates the hypothalamus-pituitary-testis axis, thus increasing FSH and testosterone secretion and testicular size [35]. It can, therefore, be deduced that the improved reproductive performance of rams determined an earlier onset of reproductive activity in young ewes.

## 5. Conclusions

Data from the present study confirmed that melatonin treatment in rams, in addition to their replacement with novel rams: (1) increased fertility rate in ewe lambs, (2) reduced the age at which first mating occurred in ewe lambs, and (3) induced a concentration of the lambing period. Furthermore, as the study provided evidence of these results being achieved via melatonin treatment of the rams alone, farmers could henceforth make considerable economic savings by eliminating the cost of melatonin treatment in all flock ewes. This study, therefore, provides a first exploration of a novel technique that breeders could utilize to reduce the age of onset of first mating and shorten the period from weaning to first lambing.

## Figures and Tables

**Table 1:** Number, least square means (±SEM) for weight and age of ewes in each group at the moment of ram introduction. The distribution of ewes within groups was performed by ANOVA (*p* > 0.05).

Group	N. Ewes	Weight (kg)	Age (Days)
RMR ^1^	100	31.91 ± 1.30	239.80 ± 10.32
RM ^2^	100	32.23 ± 1.38	240.15 ± 10.14
RCR ^3^	100	32.62 ± 1.36	240.33 ± 10.53
RC ^4^	100	32.84 ± 1.32	239.32 ± 10.41

^1^ RMR = ewes mated with rams treated with melatonin and replaced every 10 days; ^2^ RM = ewes mated with melatonin-treated rams; ^3^ RCR = ewes mated with untreated control rams replaced every 10 days; ^4^ RC = ewes mated with untreated control rams.

**Table 2:** Proportion of mated ewes, pregnant ewes, lambed ewes, and ewes that did not lamb in the four groups. The results were analyzed by Chi-square test.

Group	Number	Mated	Pregnant	Lambed	Not Lamb
RMR ^1^	100	0.90 ^c^	0.86 ^c^	0.83 ^c^	0.17 ^a^
RM ^2^	100	0.77 ^b^	0.74 ^b^	0.72 ^b^	0.28 ^b^
RCR ^3^	100	0.68 ^a^	0.65 ^a^	0.62 ^a^	0.38 ^c^
RC ^4^	100	0.67 ^a^	0.64 ^a^	0.61 ^a^	0.39 ^c^

^1^ RMR = ewes mated with rams treated with melatonin and replaced every 10 days; ^2^ RM = ewes mated with melatonin-treated rams; ^3^ RCR = ewes mated with untreated control rams replaced every 10 days; ^4^ RC = ewes mated with untreated control rams. Different lower-case letters within columns (a, b, c) indicate differences for *p* ≤ 0.05.

**Table 3:** Least square means (±SEM) for litter size and DRIL of the levels of treatment effect, replacement effect, and interaction between treatment and replacement effect with significance of contrasts between least square means.

Factor	Level	N. Ewes	Litter Size	*p* Value	DRIL ^9^	*p* Value
Treatment	Treated ^1^	200	1.24 ± 0.03	0.064	173.31 ± 9.94	0.009
	Untreated ^2^	200	1.17 ± 0.02		185.93 ± 9.95	
Replacement	With replacement ^3^	200	1.22 ± 0.02	0.081	176.18 ± 10.98	0.032
	Without replacement ^4^	200	1.19 ± 0.03		182.18 ± 11.83	
Treatment by replacement	RMR ^5^	100	1.27 ± 0.03	0.062	170.95 ± 8.11 ^a^	0.001
	RM ^6^	100	1.23 ± 0.01		176.02 ± 11.12 ^a^	
	RCR ^7^	100	1.18 ± 0.01		182.71 ± 10.61 ^b^	
	RC ^8^	100	1.14 ± 0.02		189.32 ± 7.96 ^b^	

^1^ Treated = ewes mated with melatonin-treated rams; ^2^ Untreated = ewes mated with untreated control rams; ^3^ With replacement = ewes mated with rams replaced every 10 days; ^4^ Without replacement = ewes mated without rams replaced; ^5^ RMR = ewes mated with rams treated with melatonin and replaced every 10 days; ^6^ RM = ewes mated with melatonin-treated rams; ^7^ RCR = ewes mated with untreated control rams replaced every 10 days; ^8^ RC = ewes mated with untreated control rams; ^9^ DRIL = distance in days from ram introduction to lambing. *p* value ≤0.05 was considered statistically significant. Different superscripts within a column (a, b) indicate difference for *p* ≤ 0.01.

## Data Availability

The data presented in this study are available on request from the corresponding author. The data are not publicly available to preserve privacy of the data.
